# The association between BDNF levels and risperidone-induced weight gain is dependent on the *BDNF* Val66Met polymorphism in antipsychotic-naive first episode schizophrenia patients: a 12-week prospective study

**DOI:** 10.1038/s41398-021-01585-3

**Published:** 2021-09-04

**Authors:** Jiahong Liu, Pingping Wang, Leilei Sun, Xiaoni Guan, Meihong Xiu, Xiangyang Zhang

**Affiliations:** 1 The Affiliated Kangning Hospital of Wenzhou Medical University, Wenzhou, China; 2 Neurology Department, Xuan Wu Hospital of Capital Medical University, Beijing, China; 3 Qingdao Mental Health Center, Qingdao University, Qingdao, China; 4 Peking University HuiLongGuan Clinical Medical School, Beijing HuiLongGuan Hospital, Beijing, China; 5 CAS Key Laboratory of Mental Health, Institute of Psychology, Chinese Academy of Science, Beijing, China

**Keywords:** Schizophrenia, Predictive markers

## Abstract

A growing number of studies have shown that brain-derived neurotrophic factor (BDNF) is associated with weight gain during antipsychotic treatment in schizophrenia patients. However, there is still a lack of research results in the initial stage of antipsychotic treatment. This study aimed to evaluate the relationship between weight gain caused by risperidone monotherapy for 12 weeks and BDNF level in antipsychotic-naive and first-episode (ANFE) patients with schizophrenia, and we hypothesize that this may depend on *BDNF* Val66Met gene polymorphism. In a 12-week longitudinal trial, 225 ANFE patients were enrolled and treated with risperidone. Body weight was measured at baseline and during the 12-week follow-up. After treatment, the average weight of ANFE patients increased by 2.6 kg. Furthermore, we found that in patients with Val/Val genotype, the increase in serum BDNF levels was negatively correlated with risperidone-induced weight gain (*r* = −0.44, *p* = 0.008). Regression analysis showed that the baseline BDNF level was a predictor of weight gain after treatment (*β* = −0.45, *t* = −3.0, *p* = 0.005). Our results suggest that the BDNF signaling may be involved in weight gain caused by risperidone treatment. Furthermore, the negative association between weight gain and increased BDNF levels during risperidone treatment in ANFE schizophrenia depends on the *BDNF* Val66Met polymorphism.

## Introduction

Atypical antipsychotics are the first-line treatment strategy for patients with schizophrenia (SZ), which can alleviate clinical symptoms [1]. However, more and more researchers have found that atypical antipsychotics can cause prominent side effects, such as weight gain, impaired glucose tolerance, and metabolic syndrome [2]. As one of the main side effects of antipsychotics, weight gain has a profound impact on treatment compliance, quality of life, psychological adaptation, and self-esteem, which in turn is superimposed on the challenges associated with SZ diagnosis [3, 4]. Studies in the general population have shown that individuals have different susceptibility to obesity, and genetics is also involved in the development of obesity [5]. Individual differences in weight gain caused by antipsychotics have also been found in various studies of SZ, but this is true even the same antipsychotics are used. Several studies have found that some candidate genes are associated with weight gain induced by antipsychotics, such as brain-derived neurotrophic factor *(BDNF), FTO*, Dopamine receptor D_2_ *(DRD*_*2*_*)*, serotonin 2A receptor *(HTR2A)*, serotonin 2C receptor *(HTR2C)*, methylenetetrahydrofolate reductase *(MTHFR)* and pro-inflammatory cytokine [6–8]. These genetic variations contribute to differences in energy expenditure, thermal effects of food, and energy consumption during exercise. However, the exact mechanism of antipsychotic-induced weight gain in antipsychotic-naive and first-episode (ANFE) SZ patients remains unclear.

BDNF is widely expressed in the hippocampus and hypothalamus of the central nervous system [9]. Based on its widespread expression in hypothalamus (the center of appetite), researchers have proposed a hypothesis that BDNF and its tyrosine kinase receptor may be closely related to eating behaviors and energy balance, and further affect obesity [10, 11]. Indeed, preclinical studies have revealed that the BDNF signaling pathway is involved in the control of food intake, activity, resting metabolism rate, body weight, and obesity [11]. Heterozygous *BDNF* gene knockout mice reduce the gene expression of BDNF, and lead to increased levels of circulating leptin, insulin, and glucose, which are related to weight gain and obesity [12]. Central administration of BDNF can transiently suppress appetite, reverse abnormal eating behaviors and obesity, and lead to weight loss [13]. Systemic administration of BDNF can decrease food intake, normalize fasting blood glucose and reduce weight in obese, non-insulin-dependent diabetic C57BLKS-mice [14]. Similarly, a recent study has shown that BDNF-mediated leptin signaling can regulate the plasticity of the sympathetic structure of adipose tissue through a top-down neural pathway, which is essential for energy homeostasis [15]. Further, reduced serum BDNF levels may be related with weight gain in female patients with SZ under long-term antipsychotic treatment [16]. In total, all these studies support that BDNF plays an important role in the regulation of body weight and eating behavior.

Previous genome-wide association study (GWAS) showed that the rs6265 polymorphism in the *BDNF* gene was closely related to obesity [17]. Moreover, in the general population from newborns, adults to ages, studies have reported that *BDNF* gene is correlated with energy, carbohydrate intake, physical activities, behavioral characteristics, BMI, and obesity [18–22]. Studies have demonstrated that *BDNF* genetic variants may confer susceptibilities to weight gain and obesity induced by antipsychotic treatment [23–25], but with inconsistent results. For example, Zhang et al. found that the Met/Met genotype of the *BDNF* Val66Met polymorphism has a significant effect on body mass index (BMI) gain in male SZ patients treated with long-term antipsychotic drugs [26]. Fonseka reported that Val/Val was associated with greater body weight gain induced by antipsychotics [27]. Tsai et al. failed to find the relationship between the Val66Met polymorphism and weight gain after antipsychotic treatment, but they reported a significant difference in percentage weight change in patients with different copies of haplotype GTA (rs6265-rs11030101-rs12291186) [24].

The clinical heterogeneity among those studies is influenced by a variety of confounding factors, including the type of antipsychotics, the disease phases of patients recruited in the study and genetic variants, which warrant further investigation. In order to minimize the confounding factors in our study, we recruited a large sample size of ANFE patients with SZ and treated them with risperidone monotherapy for 12 weeks. Also, all patients stayed in the hospital throughout the trial. Therefore, potential confounding variables such as diet, physical activity, and drug compliance were well controlled. This setting provided a favorable environment for investigating the possible role of BDNF in weight changes after 12 weeks of antipsychotic treatment.

In the present study, we conducted a three-month follow-up study of ANFE patients with SZ who received risperidone monotherapy. We hypothesized that serum BDNF levels were associated with the weight gain caused by risperidone treatment. We also expect to see whether the association between BDNF levels and weight gain was regulated by the *BDNF* Val66Met polymorphism.

## Subjects and methods

### Subjects

According to the criteria in the previously published literature, we recruited 225 ANFE patients with SZ [28, 29]. Inclusion criteria included: age between 16 and 45 years; disease duration <60 months; first episode of disease; no previous treatment with antipsychotics or cumulative exposure to antipsychotics <2 weeks; diagnosis of SZ according to the *Diagnostic and Statistical Manual of Mental Disorders, Fourth Edition* (DSM-IV) using the *Structured Clinical Interview for DSM-IV* (SCID). Also, 125 unrelated healthy controls were recruited from the local community through advertisements. None of them had a personal or family history of any major Axis I disorder confirmed by three clinical psychiatrists according to SCID.

All patients and controls were Han Chinese. Detailed medical information was obtained from all subjects, including demographics, sociodemographic characteristics, and medical conditions. All participants received a physical examination and laboratory testing. Individuals with unstable diabetes, cancer, infections, cerebrovascular disease, cardiovascular disease, and pregnancy were excluded. The Institutional Review Board (IRB) of the Beijing Huilongguan Hospital approved this protocol, and all participants provided written informed consent.

### Clinical treatment and assessments

Since admission, patients were treated with a flexible dose of risperidone for three months. Agitated patients were allowed to be injected with haloperidol for a short period of time (usually <1 week). Benzodiazepine was permitted to be used in patients with sleep disorders and anticholinergic drugs were permitted to be used in patients with extrapyramidal side effects. We evaluated clinical symptoms at baseline and at the end of 12 weeks. Six clinical psychiatrists assessed the psychiatric symptoms of patients through the Positive and Negative Syndrome Scale (PANSS) after a training course [30]. In addition, under the condition of wearing light clothes, no shoes, and empty pockets, the body weight after overnight fasting was measured at baseline and follow-up. The same scale was used during the trial, and the measurement of each patient was repeated twice to take the average value.

### Determination of BDNF levels and BDNF Val66Met genotyping

Blood samples were collected following an overnight fast. The levels of serum BDNF were measured by enzyme-linked immunosorbent assay (ELISA) [31], which were performed by a technician who was blind to the clinical status. A standard protocol was used to extract genomic DNA (Promega, USA). The BDNF Val66Met polymorphism was genotyped as reported in our previous study [32]. Genotyping was duplicated and carried out by the same research assistant, who was blinded to the status of the subjects.

### Statistical analysis

Demographics, clinical characteristics, weight, and BMI were compared by analysis of variance (ANOVA) or *X*^2^ tests between patients and healthy controls at baseline. The Hardy-Weinberg equilibrium (HWE) was examined in healthy controls and patients. *X*^2^ test was used to analyze the difference in the allele and genotype frequencies of Val66Met polymorphism between patients and controls. At baseline, ANCOVA was performed to compare the BDNF level between controls and patients. The covariates in the ANCOVA analysis included age, sex, smoking status, and BMI. For the main model, three BDNF genotypes and diagnostic groups (patients vs controls) were entered as fixed effects. Weight, BMI, BDNF levels, and clinical symptoms were entered as dependent variables. The genotype × diagnosis interaction term analyzed the different effects that genotype may have on the weight, BMI, and symptoms between the diagnostic groups. Then, Pearson product-moment correlation analysis was performed to investigate the relationship between BDNF levels and bodyweight or BMI. Bonferroni correction was used to adjust multiple comparisons.

Lastly, at follow-up, a multiple linear regression analysis with weight gain as an independent variable was carried out to detect whether baseline BDNF levels or changes in BDNF may predict the weight gain induced by risperidone across the three genotype groups. Moreover, as described in previous studies [33], patients with a weight gain of 7% or more were classified into the WG group and non-WG group. The last observation carried forward (LOCF) analysis was carried out for patients who dropped out after the second month. A logistic regression analysis was performed to examine the relationship between BDNF genotypes and weight gain. The covariates in these regression models included age, age of onset, sex, smoking status, and duration of illness.

## Results

### Baseline weight, BDNF Val66Met genotype, and BDNF levels

There was no significant difference in the *BDNF* Val66Met genotype and allele distribution between the patients and healthy controls (*X*^2 ^= 1.2, df = 2, *p* = 0.54; and *X*^2 ^= 0.001, df = 1, *p* = 0.97, respectively). Also, there was no association between Val66Met genotype and baseline weight or BMI in controls and patients (all *p* > 0.05). Compared with controls, SZ patients showed lower BDNF levels (9.5 ± 3.9 vs 11.8 ± 2.5 ng/ml, *p* < 0.001) and BMI (21.4 ± 3.5 vs 23.5 ± 4.1, *p* = 0.001, Table 1) when controlling for sex, age, smoking status and years of education. No difference in BDNF level was found between the three genotypes both in patients and controls (all *p* > 0.05). Also, no significant association was found between baseline weight or BMI and BDNF levels in patients (all *p* > 0.05).

**Table 1:** Demographic characteristics, clinical data, and BDNF levels in antipsychotics-naive first-episode (ANFE) patients with schizophrenia (SCZ) and healthy controls.

Variable	ANFE patients (*n* = 225)	Healthy controls (*n* = 125)	*p* value
Gender(male/female)	124/101	77/48	0.26
Age (years)	27.9 ± 9.3	27.6 ± 7.0	0.90
Education (years)	9.3 ± 3.9	10.3 ± 3.1	0.01
Smoker (*n*, %)	64 (28.4)	44 (35.2)	0.23
Weight (kg)	59.4 ± 11.7	66.7 ± 13.9	<0.001
BMI (kg/m^2^)	21.4 ± 3.5	23.5 ± 4.1	0.001
Age of onset (years)	26.2 ± 9.2		
BDNF(ng/ml)	9.5 ± 3.9	11.8 ± 2.5	<0.001
*BDNF* Val66Met			0.54
Met/Met	55	34	
Val/Met	112	54	
Val/Val	58	36	

*BMI* body mass index; *PANSS* the positive and negative syndrome scale; *BDNF* brain-derived neurotrophic factor.

### Weight gain and BDNF Val66Met genotype in patients

After 12 weeks of risperidone treatment, the average weight gain and BMI increase were 2.6 ± 3.7 kg and 1.0 ± 1.3 kg/m^2^. We investigated whether *BDNF* Val66Met genotype predicted weight gain after risperidone treatment. ANCOVA analysis showed that there was no difference in the changes in weight and BMI between genotype groups (*F* = 1.4, *p* = 0.26 for weight; *F* = 1.2, *p* = 0.30 for BMI) (Table 2). There was a significant association between weight gain and age (*r* = −0.17, *p* = 0.01) or baseline BMI (*r* = −0.36, *p* < 0.001), which was controlled in the regression analysis. Linear regression analysis revealed that there was no significant association between Val66Met genotype and weight gain induced by risperidone treatment after controlling for age, baseline BMI and reduction in PANSS total score. If the patient’s weight gain exceeded 7% of the baseline weight, we defined the patient as the weight gain (WG) group, and the other patients as the non-weight gain (non-WG) group [34]. Logistic regression analysis also showed no significant association between Val66Met genotype and weight gain.

**Table 2:** Changes of BDNF levels and clinical characteristics across three genotypes in ANFE patients with schizophrenia.

Variable	Val/Val *n* = 58	Val/Met *n* = 112	Met/Met *n* = 55	F (*p*)
*Baseline*				
Weight (kg)	60.9 ± 11.2	59.5 ± 12.3	57.8 ± 10.7	1.0 (0.37)
BMI (kg/m^2^)	21.8 ± 3.0	21.4 ± 3.7	21.0 ± 3.5	0.7 (0.50)
BDNF (ng)	9.0 ± 3.9	9.7 ± 3.5	9.7 ± 4.6	0.6 (0.56)
Age (yrs)	31.1 ± 10.2	26.9 ± 9.0	26.5 ± 8.2	5.0 (0.01)
Education (yrs)	9.0 ± 4.2	9.7 ± 3.7	8.8 ± 3.7	1.4 (0.25)
Onset age (yrs)	29.1 ± 10.4	25.4 ± 8.9	24.6 ± 7.9	4.3 (0.01)
*Changes at 12-week follow-up*				
Weight gain (kg)	2.3 ± 3.4	2.6 ± 3.4	3.0 ± 4.5	0.5 (0.60)
BMI gain (kg/m^2^)	0.8 ± 1.2	1.0 ± 1.2	1.1 ± 1.6	0.4 (0.68)
BDNF levels (ng)	0.3 ± 4.3	0.4 ± 4.4	1.7 ± 9.7	0.7 (0.52)
PANSS total score	28.8 ± 21.1	23.3 ± 17.0	25.1 ± 18.3	1.6 (0.20)
P subscore	12.2 ± 7.1	9.7 ± 6.1	9.5 ± 6.3	3.2 (0.04)
N subscore	3.9 ± 7.3	4.2 ± 5.9	5.5 ± 5.7	1.0 (0.37)
G subscore	12.8 ± 10.5	9.6 ± 9.0	10.7 ± 9.5	2.0 (0.13)

*P subscore* positive subscore, *N subscore* negative subscore, *G subscore* general psychopathology subscore.

### Association between BDNF level changes, genotype, and weight gain

After treatment, the patients showed non-significant increase in BDNF levels (*p* = 0.22). Correlation analysis showed that there was no significant association between changes in BDNF levels and weight gain after treatment (*p* > 0.05). However, we found that increased BDNF levels were correlated with weight gain in Val/Val homozygote patients (*r* = −0.44, *p* = 0.008). This association remained significant after further regression analysis was performed to control for the covariates (*β* = −0.31, *t* = −2.4, *p* = 0.022).

### Baseline BDNF levels, genotype, clinical variables, and weight gain

There were significant differences in age, onset age, and reduction in positive symptoms across the *BDNF* genotypes in ANFE patients with SZ (*F* = 5.0, *p* = 0.01; *F* = 4.3, *p* = 0.01; *F* = 3.2, *p* = 0.04). No association was found between baseline BDNF levels and psychotic symptoms and clinical variables (all *p* > 0.05). Correlation analysis showed no association between baseline BDNF level or *BDNF* genotype and risperidone-induced weight gain, even stratified by sex (all *p* > 0.05). In order to explore whether weight gain was associated with baseline BDNF levels between genotype groups, further regression analysis was performed across the patients’ three BDNF genotypes. In the regression model, weight gain was an independent variable, and age, years of education, smoking status, and decrease of PANSS total score were dependent variables. The results revealed that there was a significant association between the baseline BDNF levels and weight gain induced by risperidone treatment in patients with Val/Val genotype (*β* = −0.45, *t* = −3.0, *p* < 0.01).

## Discussion

The main findings of our study were as follows. First, after 12 weeks of risperidone monotherapy, the weight of ANFE patients with SZ was significantly increased from baseline. Second, in patients with Val/Val genotype, there was a significant association between increased BDNF levels and weight gain induced by risperidone. Third, the baseline BDNF level was a predictor of risperidone-induced weight gain only in ANFE patients with Val/Val. At present, atypical antipsychotics are the first-line drugs for the treatment of SZ. Recently, the problem of weight gain caused by drugs has received more and more attention. In this large SZ clinical study of ANFE patients, we found that 12 weeks of treatment with risperidone significantly increased body weight of the patients, suggesting that atypical antipsychotic treatment can induce weight gain in the early stage of treatment.

The weight gain of SZ patients after antipsychotic treatment was complicated by many potential confounders in the environment, which were all controlled in our analysis. Moreover, considering that previous studies have shown that weight gain is closely related to the reduction of clinical symptoms of SZ, we conducted an analysis to control for the decrease of PANSS subscores. Our findings are consistent with most studies suggesting that short-term treatment with risperidone may induce weight gain in ANFE SZ patients [35]. Overall, studies support that weight gain exists in the early stage after risperidone treatment even when various environmental risk factors were well controlled. Studies in the general population have significantly shown that BDNF is correlated with appetite regulation, energy homeostasis, and obesity [8]. The relationship between BDNF serum levels or genetic variations and weight gain after risperidone treatment has also attracted special interest in previous studies, although the results of these studies were inconsistent in SZ [8, 16, 24, 36, 37]. In this study, we found that after 3 months of risperidone treatment, the increase in BDNF level or rs6265 polymorphism was not correlated with weight gain. Consistent with our study, a large cohort study revealed that weight gain was not associated with Val66Met, but was significantly associated with the Val66Met-rs1519480 G/A haplotype [25]. In a cohort study using risperidone, olanzapine or clozapine, Tsai et al. found a strong association between weight gain and Val66Met-rs11030101, but not with Val66Met [24]. Bonaccorso et al. found that the *BDNF* Val66Met genotype was correlated with weight gain after 12 months of risperidone treatment, and Met carriers increased their bodyweight and BMI at 6 months instead of 3 months, suggesting that the Val66Met variant can only predict long-term accumulated effects of antipsychotics. Interestingly, in this study, another finding of a nonsignificant association between weight gain and changes of BDNF level during the first 3 months of risperidone treatment in ANFE patients provided further evidence that BDNF was not related with a short-term effect, but with a long-term accumulated effect of risperidone. Altogether, the current research supports that in the first 3 months of treatment in ANFE patients, serum BDNF levels and Val66Met polymorphism were not associated with weight gain induced by risperidone. Interestingly, we found that *BDNF* Val66Met polymorphism affected the relationship of BDNF level with risperidone-induced weight gain in SZ patients. In those patients with Val/Val homozygote, we found that the increase in BDNF level was negatively correlated with the increase in weight, suggesting that the relationship between weight gain and BDNF levels only existed in a subset of patients. To our best knowledge, this is the first study to investigate the relationship between BDNF levels, genotypes, and weight gain in ANFE SZ patients treated with risperidone. How Val66Met polymorphism influences the level of BDNF and further participates in weight gain remains unknown. Preclinical studies have shown that BDNF and its receptors are widely expressed in the hypothalamus and are closely related to eating behavior, food consumption, and weight control [13, 38]. Moreover, it has been reported that BDNF regulates the mesolimbic dopamine system in mice, which is essential for reward behavior [39]. However, in this study, we found that the serum levels of BDNF in SZ patients were not associated with the Val66Met genotype [40]. Therefore, the underlying mechanism of Val/Val genotype in weight gain is not through affecting the BDNF levels, but through affecting the function of BDNF. Neurons with mutant Val66Met genotype have lower secretion and fail to be located in synapses, indicating that Val66Met plays an important role in the intracellular transport and activity-dependent secretion of BDNF in certain brain regions [9].

Therefore, one possible explanation for these results is that patients with the Val/Val genotype have higher secretion and improved transport of BDNF protein than their Val/Met and Met/Met counterparts. In addition, antipsychotic-induced weight gain is a polygenetic disorder with a complex gene interaction network of multiple genes, such as HTR2C gene, insulin-induced gene 2 (INSIG2), leptin, alpha 2A adrenergic receptor, cannabinoid receptor 1 (CNR1), and melanocortin 4 receptor (MC4R) [41–45]. We speculate that the epistasis or multilocus interactions, such as BDNF and other key genes related to weight gain are involved in the relationship between BDNF and weight. However, due to the relatively small sample size of this study, we were unable to analyze the haplotypes of multiple genes to rule out the possibility that other risk genes have nominal effects on this association across ethnic groups.

Several limitations should be noted in this study. First, weight gain induced by antipsychotics is regulated by multiple genetic polymorphisms in several candidate genes. Therefore, in future research, polygenic models, such as epistasis should be investigated in a larger sample size. Second, we assessed the level of BDNF in the serum, not in the brain. It is still uncertain whether the serum level of BDNF is correlated with the level in the central nervous system. Third, only the effect of risperidone was examined in this trial. Further research should investigate whether other antipsychotic drugs have similar findings. Fourth, we did not collect laboratory measures of metabolic functioning, such as fasting glucose, fasting lipids, and related metabolic parameters. Therefore, our findings are preliminary.

In conclusion, this study found that risperidone monotherapy for 12 weeks can significantly increase the weight of ANFE SZ patients. Although serum BDNF levels and Val66Met polymorphism are not associated with risperidone-induced weight gain, we did find that in patients with Val/Val genotype, the increase in BDNF levels and BDNF baseline levels were negatively associated with weight gain, indicating that baseline level of BDNF and Val66Met variant are predictors of weight gain in ANFE SZ patients treated with risperidone. Due to the drawbacks of this study design, a randomized, double-blind, placebo-controlled trial with a large sample size in a long-term treatment will be warranted to elucidate the relationship between weight gain, BDNF serum levels and *BDNF* gene polymorphisms.
